# Hypereosinophilic syndrome presenting as coagulopathy

**DOI:** 10.1186/s13223-022-00666-2

**Published:** 2022-03-22

**Authors:** Kestutis Aukstuolis, Jocelyn J. Cooper, Katherine Altman, Anna Lang, Andrew G. Ayars

**Affiliations:** 1 Division of Allergy and Infectious Diseases, Department of Medicine, University of Washington, Seattle, WA USA; 2 Division of Internal Medicine, Department of Medicine, University of Washington, Seattle, WA USA; 3 Northwest Asthma and Allergy Center, Redmond, WA 98052 USA

**Keywords:** Hypereosinophilic syndrome, Thrombosis, Bowel perforation, Anti-IL-5-receptor therapy, Benralizumab, Ischemic colitis, Eosinophil extracellular traps, Coagulopathy

## Abstract

**Background:**

Hypereosinophilic syndrome (HES) is an extremely uncommon group of disorders. It rarely presents with coagulopathy without cardiac involvement.

**Case presentation:**

A 33-year-old previously healthy male with no history of atopic disease presented with abdominal pain, hematochezia, peripheral eosinophilia as high as 10,000 eos/µL, right and left portal vein, mesenteric, and splenic vein thrombi with ischemic colitis resulting in hemicolectomy and small bowel resection. Despite an extensive workup for primary and secondary etiologies of hypereosinophilia by hematology/oncology, infectious disease, rheumatology and allergy/immunology, no other clear causes were identified, and the patient was diagnosed with idiopathic HES. His eosinophilia was successfully treated with high-dose oral corticosteroids (OCS) and subsequently transitioned to anti-IL-5-receptor therapy with benralizumab. He has continued this treatment for over a year with no recurrence of eosinophilia or thrombosis while on benralizumab.

**Conclusion:**

In patients with an unexplained coagulopathy and eosinophilia, eosinophilic disorders such as HES should be considered. Corticosteroid-sparing agents, such as benralizumab show promise for successfully treating these patients.

## Background

Hypereosinophilic syndrome (HES) is a group of disorders defined as elevated peripheral blood eosinophil count ≥ 1500 eosinophils per microliter (eos/µL) on two occasions ≥ 1 month apart with organ dysfunction attributable to eosinophilia [1]. These disorders are rare, thought to have a prevalence rate between 0.315 and 6.3 per 100,000 in the United States [2]. Types of HES include primary (clonal/neoplastic), secondary (reactive), or undetermined significance, also known as idiopathic HES [3]. Idiopathic HES is a diagnosis of exclusion following workup for primary or secondary causes.

HES typically presents with symptoms of weakness and fatigue, cough, dyspnea, myalgia, angioedema, rash, or fever [3]. The most common organ systems involved with presentation of HES are dermatologic (69%), pulmonary (44%), and gastrointestinal (38%) [3]. Although up to 20% of HES patients have related cardiac disease, only 6% of cases have this at presentation [3]. Rheumatologic, and neurologic manifestations have also been well-established [1]. Thromboembolic events are thought to occur in 25% of patients with cardiac involvement of HES, which has previously been attributed to endocardial damage leading to mural thrombi [3, 4]. More recently, it has been demonstrated that eosinophils play a direct role activating platelets and promoting thrombus formation [6].

Treatment of HES patients with thrombi have previously include corticosteroids, anticoagulants, and antiplatelet agents, which have many risks when used long term [3]. Recently, anti-interleukin-5 (anti-IL-5) and anti-IL-5-receptor antibody therapies have shown promise as steroid-sparing treatment for HES [5]. Here we describe a case of idiopathic HES in a previously healthy patient who presented with mesenteric, portal, and splenic vein thromboses and ischemic colitis, who was treated with benralizumab.

## Case presentation

A 33-year-old previously healthy male with no history of travel presented with abdominal pain, hematochezia, peripheral eosinophilia (6200 eos/µL), mesenteric, splenic, and portal vein thromboses resulting in ischemic colitis. Approximately 1 month prior to his hospitalization he had a self-limiting illness lasting five days with fever, nonproductive cough, myalgias, chills, and sweats. After resolution of this illness, he developed right upper thigh pruritus and bruising along with polyarthralgias which was unresponsive to prednisone. He did not have any rashes or angioedema. He was admitted for four days to an outside hospital after significant thrombocytopenia (20,000/μL) and eosinophilia (6200 eos/µL) were noted on CBC (Table 1).

**Table 1:** Initial laboratory findings

	Value	Reference range
WBC	15.3 K/μL	4–11 K/μL
RBC	5.42 M/μL	4.31–5.77 M/μL
HGB	15.6 g/dL	13.2–17.5 g/dL
HCT	45.30%	38.9–49.9%
MCV	83.6 fL	80–100 fL
MCH	28.8 pg	27.8–33.8 pg
MCHC	34.4 g/dL	31.5–36.5 g/dL
RDW	11.90%	11.5–14.2%
Platelets	20 K/μL	150–400 K/μL
MPV	12.5 fL	8.5–12.4 fL
Neutrophils %	41.4	
Immature granulocyte %	0.4	
Lymphocytes %	12.3	
Monocytes %	5.3	
Eosinophils %	40.2	
Basophils %	0.4	
Neutrophil number	6.3 K/μL	1.5–8 K/μL
Immature granulocyte number	0.1 K/μL	0–0.1 K/μL
Lymphocyte number	1.9 K/μL	1–3.5 K/μL
Monocyte number	0.8 K/μL	0.2–1 K/μL
Eosinophil number	6.2 K/μL	0–0.5 K/μL
Basophil number	0.1 K/μL	0–0.2 K/μL
Prothrombin time	17.1 s	11.8–14.9 s
INR	1.4	0.9–1.1
Fibrinogen	197 mg/dL	200–450 mg/dL
Fibrin Split Products (FDP-Latex)	80 μg/mL	< 5 μg/mL

Upon admission he was thought to have idiopathic thrombocytopenia purpura (ITP) and treated with intravenous immunoglobulin (IVIG), dexamethasone, and transfused one unit of platelets. Computed tomography (CT) showed nonocclusive right and left portal vein, mesenteric, and splenic vein thrombi. Bone marrow biopsy showed increased eosinophils accounting for 25% of granulocytes and 20% of total cells with no significant immunophenotypic abnormalities of myeloid cell populations and no abnormal B cell, T cell, or plasma cell populations identified. He was discharged from the outside hospital on warfarin after having completed a course of dexamethasone 40 mg daily for four days. His eosinophil count at discharge was 0 eos/µL.

Two weeks later, he again presented to the outside hospital after having severe abdominal pain, hematochezia, and fevers. Upon admission, noted to have eosinophil count of 5700 eos/µL. Endoscopy and subsequent pathology showed patchy eosinophilic infiltrates from esophagus through the colon along with evidence of ischemia. His eosinophil count continued to climb to a high of 10,000 eos/μL. He was placed on IV corticosteroids. Due to ongoing abdominal pain and concern for possible HES, he was transferred to our hospital for further evaluation and management.

Upon admission, he developed worsening abdominal pain and contrast CT abdomen/pelvis revealed necrotic ascending colon and small bowel with ascending colon perforation, prompting emergent laparotomy, right hemicolectomy, and segmental small bowel resection. Pathology of the transverse colon showed increased eosinophils in the laminal propria with eosinophilic cryptitis along with eosinophils in the sigmoid colon. Additionally, pathology revealed thrombi involving submucosal, subserosal, and mesenteric elastic arteries and arterioles. No parasitic organisms were identified on any of the pathology slides. His troponin-I was negative (< 0.03 ng/mL) and his EKG showed normal sinus rhythm with no ST segment elevation or depression. His echocardiogram was normal. His chest radiographs were normal. Abdominal CT showed normal-sized spleen.

Primary and secondary causes of eosinophilia were excluded by hematology/oncology, infectious disease, and rheumatology (Table 2).

**Table 2:** Evaluation for primary and secondary causes of eosinophilia

Vitamin B12	> 1500 pg/mL (range 180–914 pg/mL)
Serum tryptase	7.1 ng/mL
Serum IgE	444 IU/mL
CMV quantitative PCR	None detected
Cryptosporidium Ag, Blood	Negative
Brucella antibody titer	< 1:80
Q Fever IgG Phase I Screen	Negative
Q Fever IgG Phase II Screen	Negative
Q Fever IgM Phase I Screen	Negative
Q Fever IgM Phase II Screen	Negative
Stool ova and parasites	Negative
Stool clostridioides difficile DNA detection	Negative
Blood culture	Negative
Stool culture	No enteric pathogens isolated
Anti-HBc IgM	Non-reactive
HBsAg	Non-reactive
Anti-HCV	Non-reactive
Anti-HAV IgM	Non-reactive
HIV antigen	Not detected
HIV-1 and HIV-2 antibodies	Not detected
Cardiolipin IgG	Negative
Cardiolipin IgM	Negative
Cardiolipin IgA	Negative
Lupus anticoagulant	Not detected
Glycoprotein I IgG	Negative
Glycoprotein I IgA	Negative
Glycoprotein I IgM	Negative
ANCA	Negative
ANA	Negative
Rheumatoid factor	Negative
Chromatin antibody	Negative
Sm/RNP antibody	Negative
DsDNA antibody	Negative
Jo-1 antibody	Negative
Scl-70 antibody	Negative
Ss-B antibody	Negative
Ss-A antibody	Positive (1.5 U)

Evaluation also excluded genetically based myeloproliferative disorders and malignancies (Table 3).

**Table 3:** Evaluation of genetically based myeloproliferative disorders, malignancies, and primary immune deficiencies

JAK2 V617F mutation	Not detected
CALR mutation	Not detected
JAK2 Exon 12 mutation	Not detected
MPL Exon 10 mutation	Not detected
CSF3R Exon 14/17 mutation	Not detected
Hereditary hemochromatosis DNA mutation analysis	No mutations detected
SCFD2, LNX, FIP1L1, CHIC2, PDGFRA/KIT on Chromosome 4q12	No rearrangements detected on interphase fluorescence in situ hybridization (IFISH)
PDGFRB	No rearrangements detected on IFISH
FGFR1	No rearrangements detected on IFISH
BCR/ABL p210 Interpretation	No BCR/ABL b2:a2 (e13:a2) or b3:a2 (e14:a2) transcripts detected
University of Washington Heme Gene Panel (ASXL1, CBL, CSF3R, DNMT3A, EZH2, FBXW7, FGFR1, FLT3, GATA1, GATA2, HRAS, IDH1, IDH2, JAK2, KIT, KMT2A, KRAS, MAP2K1, MPL, MYD88, NOTCH1, NPM1, NRAS, PDGFRA, PHF6, PTEN, RB1, RUNX1, SF3B1, SRSF2, STAG2, STAT3, TET2, TP53, U2AF1, WT1, ZRSR2)	No pathogenic mutations detected

The patient was diagnosed with idiopathic HES and treated with 60 mg of IV methylprednisolone daily while continuing anticoagulation therapy as an inpatient. He was transitioned to 60 mg daily of prednisone prior to discharge while maintaining an eosinophil count of 0 eos/µL with no further evidence of coagulopathy. Shortly after discharge, benralizumab was started at 30 mg subcutaneously every 4 weeks for three doses then every 8 weeks. As benralizumab was started, he was simultaneously weaned off corticosteroids over the course of several months while maintaining peripheral eosinophil count of 0 eos/µL. After remaining asymptomatic for 10 months while on benralizumab, anticoagulation therapy was discontinued. His eosinophils have remained at 0 eos/µL and has had no further evidence of coagulopathy after 1 year of therapy with benralizumab. He will continue benralizumab indefinitely.

## Discussion and conclusions

Eosinophils have been shown to play a direct role in atherosclerotic plaque formation and thrombosis. Recent studies have shown that eosinophils are activated in atherosclerosis and support plaque formation by increasing von Willebrand factor exposure and platelet adhesion to the endothelium [6]. They are also recruited during arterial thrombosis via integrins and serve to stabilize thrombi through platelet interaction [6]. Eosinophils are stimulated by platelets to form eosinophil extracellular traps which then activate platelets through major basic protein [6]. A similar, well-studied mechanism results in recruitment of neutrophils and monocytes during plaque rupture which contribute to thrombosis via neutrophil extracellular traps [6].

With the patient’s significant eosinophilia, it is not completely clear why he presented with thromboses in his gastrointestinal tract as opposed to developing cardiac complications, as is often seen with idiopathic HES. Based on previous studies, he was at risk of developing cardiac complications, given his thrombocytopenia and elevated vitamin B12 [7]. However, it is thought that eosinophil-mediated cardiac disease evolves in three stages [8]. The first stage involves acute necrosis and damage to the endocardium and myocardium. The second is a thrombotic stage, which is then followed by a third fibrotic stage [8]. On average, the thrombotic stage can be found in patients who have had 10-months of eosinophilia [8]. Prior to his hospitalization, it is unclear how long he had eosinophilia. It could be postulated that his disease may not have been present long enough to progress to this stage. In the near future, we may better understand why cardiac involvement is seen in certain HES patients, as models are being developed to investigate this phenomenon [9].

While corticosteroid therapy is first line for idiopathic HES, newer biologic medications have shown promise in reducing eosinophilia and clinical symptoms of HES. Mepolizumab, an anti-IL-5 agent, was approved by the FDA in September 2020. In a phase III trial, Mepolizumab reduced flares in treatment-refractory FIP1L1-PDGFRA–negative HES by 50% in comparison to placebo group [10]. Reslizumab, another anti-IL-5 agent, has shown some promise in treating HES; however, its use has been limited to a pilot study and case reports [11]. Benralizumab is an anti-IL-5 receptor agent approved for severe eosinophilic asthma. In a recent phase II trial, it was found to be effective in reducing eosinophilia in treatment-refractory, PDGFRA–negative HES [12]. Benralizumab was used to take advantage of its dosing regimen, opportunity for self administration, as the patient lives farther away from specialty care.

There are no guidelines regarding duration of anticoagulation therapy for patients with HES and thrombosis once the eosinophilia has been addressed. Several case reports have described recurrence of thromboembolic events even with adequate anticoagulation. Given evidence that eosinophils play a direct role in thrombosis, targeting eosinophils may prevent further thrombotic complications. Once eosinophil levels have normalized, discontinuation of anticoagulation could be considered if thrombi have resolved, and no other indication exists based on patient risk factors [4].

Our patient highlights the importance of having an increased index of suspicion for HES in patients presenting with eosinophilia and thrombosis. Rapid diagnosis of HES and utilization of steroid-sparing agents such as anti-IL-5 therapies could help prevent further end-organ damage, treat an underlying mechanism for associated coagulopathy, and avoid long-term side effects of corticosteroid use.

## Data Availability

Data sharing is not applicable to this article as no datasets were generated or analyzed for this case report.

## Abbreviations

HES: Hypereosinophilic syndrome
Anti-IL-5: Anti-interleukin-5
CT: Computed tomography
OCS: Oral corticosteroids
FDA: Food and Drug Administration
